# Association between polycyclic aromatic hydrocarbons exposure and metabolic dysfunction-associated steatotic liver disease in US adults

**DOI:** 10.3389/fpubh.2025.1540357

**Published:** 2025-06-11

**Authors:** Jiajun Wu, Shaoqian Cui, Xuekui Li, Xiaofei Zhang, Siqi Yang, Junhao Sun, Xiubo Jiang

**Affiliations:** ^1^Department of Epidemiology and Health Statistics, School of Public Health, Qingdao University, Qingdao, China; ^2^Qingdao Municipal Center for Disease Control and Prevention, Qingdao Institute of Prevention Medicine, Qingdao, China; ^3^Chengdu Xindu District Center for Disease Control and Prevention, Chengdu, China; ^4^Linyi People's Hospital, Linyi, China

**Keywords:** polycyclic aromatic hydrocarbons, metabolic dysfunction-associated steatotic liver disease, BKMR, WQS, NHANES

## Abstract

**Objective:**

To investigate the individual or combined effects of polycyclic aromatic hydrocarbons (PAHs) on metabolic dysfunction-associated steatotic liver disease (MASLD) in U.S. adults.

**Methods:**

We enrolled 3,130 participants aged 20 and over from the 2007–2016 National Health and Nutrition Examination Survey (NHANES) and analyzed six urinary PAH metabolites. The Poisson regression, Bayesian kernel machine regression (BKMR), and weighted quantile sum (WQS) regression models were used to assess the associations between PAHs and MASLD.

**Results:**

After adjusting for covariates, Poisson regression model showed significant associations [*RRs* (95% *CIs*)] between higher exposure quartiles of 2-hydroxynaphthalene (2-NAP) [Q2: 1.35 (1.06, 1.73); Q3: 1.67 (1.35, 2.07); Q4: 1.62 (1.23, 2.15); *p*-trend < 0.001], 2-hydroxyfluorene (2-FLU) [Q3: 1.36 (1.08, 1.70); *p*-trend = 0.073], 1-hydroxyphenanthrene (1-PHE) [Q4: 1.35 (1.03, 1.76); *p*-trend = 0.009], and 1-hydroxypyrene (1-PYR) [Q3: 1.37 (1.12, 1.69); Q4: 1.33 (1.01, 1.76); *p*-trend = 0.025] and MASLD (*p* < 0.05), compared with Q1. BKMR model exhibited a positive trend between mixed PAHs and MASLD. The WQS index constructed for six PAHs was significantly related to MASLD [*OR* (95% *CI*): 1.25 (1.06, 1.49)].

**Conclusion:**

This study suggests that exposure to PAHs, individually or in combination, may be associated with an increased risk of MASLD.

## Introduction

Nonalcoholic fatty liver disease (NAFLD) is the leading cause of chronic liver disease globally, which can eventually develop into hepatocellular carcinoma and liver failure (1). It represents the liver manifestation of a multisystem disease that is heterogeneous in its underlying causes, presentations, progression, and outcome (2). Given the imperfect nomenclature and diagnostic criteria of NAFLD, a revised definition of metabolic dysfunction-associated fatty liver disease (MAFLD) was proposed by an expert panel in 2020 (3). Unlike NAFLD, the diagnosis of MAFLD does not necessitate the exclusion of other chronic liver disorders or significant alcohol consumption. Recently, considering the Delphi consensus, the term MAFLD has been replaced with the new term metabolic dysfunction-associated steatotic liver disease (MASLD) (4). While the use of medical terminology such as “steatosis” may be seen as over medicalizing the lexicon to some extent, it avoids the pejorative and stigmatizing connotations of the term “fatty” and facilitates patients’ disclosure of their health status. Moreover, the perception among healthcare providers that “fatty liver” is an indolent condition has led to only limited success in raising disease awareness. With therapeutics on the horizon, a renewed focus on identifying “at-risk” patients, coupled with the adoption of new, more precise terminology, such as “steatotic” may increase awareness and attention to the disease (4).

MASLD emphasizes the significant impact of cardiac metabolic risk factors on the pathogenesis and progression of steatotic liver disease, and is more accurate in the identification of people who are at higher risk of type 2 diabetes mellitus (T2DM) (5). MASLD can result in the development of liver fibrosis, liver cirrhosis and hepatocellular carcinoma, and extrahepatic complications including T2DM, cardiovascular disease (CVD), renal disease and certain extrahepatic cancers (6, 7). It is estimated that the global prevalence of MASLD among adults is about 30% (8). MASLD causes significant global health and economic burden, and no specific drug treatments are currently approved (6). Therefore, there is a great need to identify risk factors for MASLD and develop prevention strategies.

Polycyclic aromatic hydrocarbons (PAHs) constitute a category of persistent, semivolatile organic pollutants featuring several fused benzene rings in various structural configurations (9). PAHs can be generated through biological processes as well as from incomplete combustion, originating from both natural and anthropogenic sources, such as forest and brush fires, or vehicle emissions and smoke, and are primarily introduced into the human body via ingestion, skin contact, and inhalation (10). Upon entering the body, PAHs are converted into hydroxylated metabolites, readily excreted in urine (11). Therefore, hydroxylated PAH metabolites in urine effectively reflect PAHs exposure (12). Certain PAHs are recognized as carcinogens, mutagens, and teratogens, representing a serious health risk (13). Prior studies demonstrated PAHs may be implicated in asthma, nerve injury, CVD, kidney and liver damage, cataracts, skin redness and inflammation, and adverse birth outcomes (14, 15).

The liver is the primary organ responsible for metabolizing PAHs and contains high levels of cytochrome P450 (CYP). PAH-induced activation of the aryl hydrocarbon receptor (AhR) regulates CYP expression (16). Abnormal activation of AhR can interfere with the estrogen signaling pathway (17), leading to oxidative stress and inflammatory responses (18, 19), which may contribute to insulin resistance (20), and ultimately result in liver damage. In a study of a nationally representative sample of U.S. adults, PAHs exposure was found to be associated with increased insulin resistance, impaired *β* cell function, and an increased prevalence of metabolic syndrome (21). A study involving Korean women found that PAHs may contribute to the pathogenesis of insulin resistance through methylation mediated inhibition of the IRS2 gene (22).

Epidemiological studies indicated negative impacts of certain PAHs on hepatic enzymes and NAFLD. A study from China demonstrated a correlation between urinary PAH metabolites and elevated levels of gamma-glutamyltransferase (GGT), ALT, and aspartate aminotransferase (AST) in adults (23). ALT and AST serve as sensitive indicators of liver cell damage, which are often used to assess liver dysfunction (24). GGT is one of the best predictors of liver mortality. The results of studies investigating the associations between PAHs and NAFLD are inconsistent. A study by the Korean National Environmental Health Survey (KoNEHS) showed that exposure to low levels of volatile organic compounds and PAHs might adversely affect the risk of NAFLD in adolescents, with 2-hydroxyfluorene (2-FLU) being the largest contributor (25). However, no significant association between 2-FLU exposure and NAFLD risk was found in a study of US adults (26). Although previous studies have primarily investigated NAFLD, the relationship between PAHs exposure and MASLD remains uncertain.

Consequently, based on data from five consecutive survey cycles of the National Health and Nutrition Examination Survey (NHANES) from 2007 to 2016, this study used statistical methods such as Poisson regression, restricted cubic spline (RCS), weighted quantile sum (WQS) regression, and Bayesian kernel machine regression (BKMR) to explore the relationship between individual and combined exposure to PAHs and the risk of MASLD, as well as differences in associations across age groups and sex.

## Methods

### Study population

**Figure 1:**
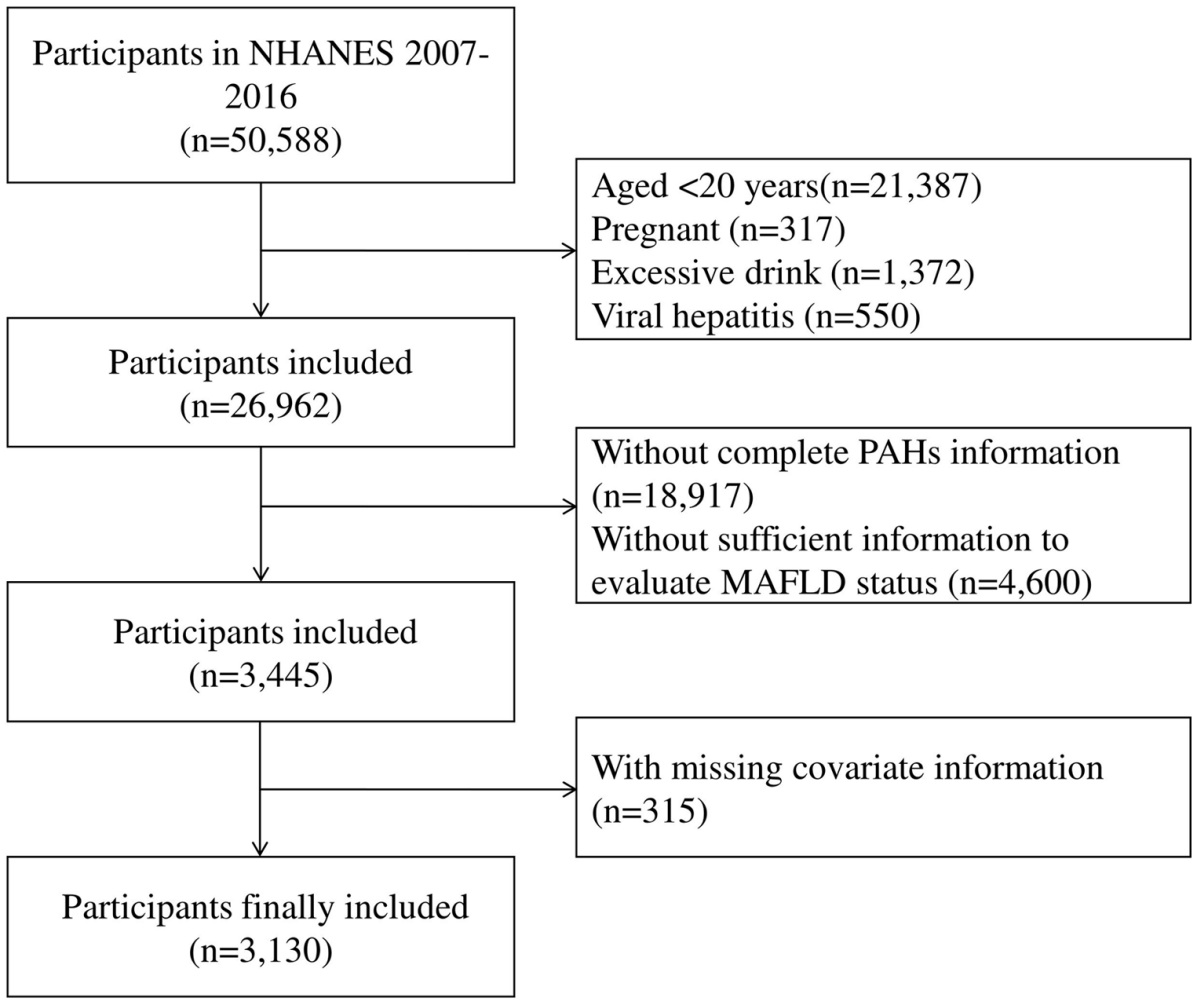
Flowchart for selecting eligible participants (National Health and Nutrition Examination Survey, United States, 2007–2016).

Data from five separate NHANES cycles (2007–2008, 2009–2010, 2011–2012, 2013–2014, and 2015–2016), a population-based survey designed to assess the health and nutritional status of the non-institutionalized U.S. population, were combined. In brief, the exclusion criteria were: (1) aged under 20 years old; (2) pregnant women; (3) excessive alcohol consumption; (4) viral hepatitis (positive for serum hepatitis B surface antigen or for serum hepatitis C antibody or RNA); (5) without complete measurements of urinary PAH metabolites; (6) without sufficient information to evaluate MASLD status; (7) with missing covariate information. In total, 3,130 participants were included in the analysis (Figure 1). All participants gave written informed consent, and the study methods were endorsed by the National Center for Health Statistics’ Research Ethics Review Board and the Centers for Disease Control and Prevention.^1^

### Measurement of PAH metabolites

Urine samples were collected from participants in NHANES using glass containers for subsequent transport to the Division of Laboratory Sciences at the National Center for Environmental Health. Monohydroxylated metabolites of PAHs were the specific analyses measured in NHANES. Because PAH metabolites have short half-lives and can be collected non-invasively, urine samples are appropriate biomarkers of recent exposure and are commonly used in biomonitoring studies (27). Previous studies have shown that a single urine sample can be representative of an individual’s normal PAHs exposure level (28). The urinary PAH metabolites investigated in this study were 1-hydroxynaphthalene (1-NAP), 2-hydroxynaphthalene (2-NAP), 3-hydroxyfluorene (3-FLU), 2-FLU, 1-hydroxyphenanthrene (1-PHE), and 1-hydroxypyrene (1-PYR). If the analytic result value was below the limits of detection (LOD), it was substituted with the LOD divided by the square root of 2. The detailed introduction can be found in the Supplementary materials.

### Definition of MASLD

In this study, the definition of MASLD was based on hepatic steatosis, no excessive alcohol consumption, no viral hepatitis, and at least one of the following five cardiometabolic risk factors: (1) body mass index (BMI) ≥ 25 kg/m^2^ or waist circumference > 94 cm for male or > 80 cm for female; (2) fasting plasma glucose (FPG) ≥ 5.6 mmol/L or 2-h post-load glucose (2hPG) ≥ 7.8 mmol/L or hemoglobin A1c (HbA1c) ≥ 5.7% or T2DM or treatment for T2DM; (3) blood pressure ≥ 130/85 mmHg or specific antihypertensive drug treatment; (4) plasma triglycerides (TG) ≥ 150 mg/dL or lipid lowering treatment; (5) plasma high density lipoprotein cholesterol (HDL-c) ≤ 40 mg/dL for male or ≤ 50 mg/dL for female or lipid-lowering treatment (4). Individuals were considered to have hepatic steatosis if their U.S. fatty liver index (USFLI) score was 30 or more (29). USFLI = e^y^/(1 + e^y^) × 100, where y = −0.8073 × non-Hispanic black + 0.3458 × Mexican American + 0.0093 × age + 0.6151 × log_e_ (GGT) + 0.0249 × waist circumference + 1.1792 × log_e_ (insulin) + 0.8242 × log_e_ (glucose) − 14.7812. If the participants belong to “non-Hispanic black” and “Mexican American,” the value of “non-Hispanic black” and “Mexican American” is 1, and if they do not belong to that ethnicity, it is 0.

### Covariates

Based on existing literature, we selected and adjusted some covariates: age, sex, race/ethnicity, education levels, marital status, poverty income ratio (PIR), the cycle of NHANES, creatinine, cotinine and physical activity. Detailed classifications are in the Supplementary material.

### Statistical analysis

Continuous variables were presented as medians (IQR, interquartile ranges), while categorical variables were expressed as counts (percentages) to describe participant characteristics. Wilcoxon rank sum tests were employed to assess discrepancies in continuous variables between MASLD and non-MASLD participants, while Chi-square tests were utilized for categorical variables. To address the non-normal distribution of the data, the urinary levels of PAH metabolites were transformed using the natural logarithm (ln). Spearman correlations were calculated between exposures.

Three multivariate Poisson regression models were developed to estimate the relative risks (*RR*s) and 95% confidence intervals (*CI*s) associated with the risk of MASLD related to exposure to PAHs. We used the number of people included in each cycle of this study as an offset. The results of the over discretization test were not significant (*p* = 1.000), indicating that Poisson regression is appropriate for the analysis. The models were fitted by using the ln-transformed PAH levels as continuous variables and further categorized into quartiles, with quartile 1 (Q1) as reference. Model 1 was adjusted for creatinine only. Based on Model 1, Model 2 was further adjusted for age, sex, race, educational level, marital status, PIR, cycle. Based on Model 2, Model 3 was further adjusted for cotinine and physical activity. In addition, we performed subgroup analysis to examine possible differences in the associations between exposure to PAHs and MASLD regarding age and sex. We then used RCS with three knots at the 5th, 50th, and 95th percentiles to determine the dose–response relationship between PAHs and MASLD in a fully adjusted model.

BKMR was employed for the analysis of the combined effect of six PAH mixtures on individuals with MASLD. BKMR is a non-parametric Bayesian variable selection framework that allows the assessment of the combined effects of chemical mixtures on outcomes (30). BKMR can effectively estimate exposure-response functions which include both nonlinear and non-additive effects, and can determine important mixture components through variable selection (31). This study used Markov chain Monte Carlo for 10,000 iterations to fit the BKMR model.

WQS regression is also a commonly utilized statistical approach for investigating the impacts of multiple chemical exposures on health outcomes and for calculating the weights of mixture components to evaluate their relative contributions (32). The WQS index is a weighted average of PAHs, each transformed into categorical variables by quantiles. A training set consisting of 40% of the data was randomly sampled, with the remaining 60% allocated for model validation, and bootstrap resampling was set to 1,000. Adjusting for the aforementioned covariates, the association between the WQS index of the mixture and MASLD was assessed in both positive and negative directions.

Sensitivity analyses were performed to assess primary results robustness. Firstly, we included the continuous variable Healthy Eating Index 2015 (HEI-2015) as a new covariate in the regression model, while retaining all original covariates. Subsequently, the binary variable (yes/no) occupational exposure (available for the 2007–2012 cycle) was used to replace HEI-2015 in the regression model. Finally, both HEI-2015 and occupational exposure were included as covariates in the model.

For the complex sampling design of NHANES, this study used appropriate weights. A two-sided *p*-value of < 0.05 was considered statistically significant. All analyses were conducted in Stata 15.0 and R soft-ware (version 4.2.3). The R package “bkmr” and “gWQS” were used to implement BKMR and WQS, respectively.

## Results

### Population characteristics

According to the inclusion criteria shown in Supplementary Figure S1, a total of 3,130 participants were enrolled from NHANES. Of these participants, 1,067 were diagnosed with MASLD, with a sample weighted prevalence rate of 31.98% (Table 1). The median age of all participants was 49 (IQR: 34–63) years, predominantly female (50.13%). Participants with and without MASLD differed significantly in age, sex, race, education level, marital status, and physical activity (*p* < 0.001).

**Table 1:** Participant characteristics by MASLD status in adults from NHANES 2007–2016 (National Health and Nutrition Examination Survey, United States, 2007–2016).

Variables	Total (*n* = 3,130)	Non-MASLD (*n* = 2063)	MASLD (*n* = 1,067)	*p*
Age (years), median (IQR)	49 (34–63)	45 (32–61)	54 (40–67)	<0.001
Sex, n (%)				<0.001
Male	1,561 (49.87)	967 (46.87)	594 (55.67)	
Female	1,569 (50.13)	1,096 (53.13)	473 (44.33)	
Race/ethnicity, n (%)				<0.001
Mexican American	478 (15.27)	220 (10.66)	258 (24.18)	
Non-Hispanic White	1,376 (43.96)	901 (43.67)	475 (44.52)	
Non-Hispanic Black	569 (18.18)	443 (21.47)	126 (11.81)	
Other	707 (22.59)	499 (24.20)	208 (19.49)	
Educational level, n (%)				<0.001
Less than high school	752 (24.03)	422 (20.46)	330 (30.93)	
High school or equivalent	685 (21.88)	436 (21.13)	249 (23.34)	
College or above	1,693 (54.09)	1,205 (58.41)	488 (45.73)	
Marital status, n (%)				<0.001
Marry/Living with partner	1917 (61.25)	1,233 (59.77)	684 (64.10)	
Widowed/Divorced/Separated	648 (20.70)	407 (19.73)	241 (22.59)	
Never	565 (18.05)	423 (20.50)	142 (13.31)	
PIR, n (%)				0.217
<1	682 (21.79)	436 (21.13)	246 (23.06)	
≥1	2,448 (78.21)	1,627 (78.87)	821 (76.94)	
Cotinine (ng/mL), median (IQR)	0.038 (0.011–3.09)	0.037 (0.011–4.63)	0.039 (0.011–2.27)	0.868
Physical activity, n (%)				<0.001
Sedentary	777 (24.82)	460 (22.30)	317 (29.71)	
Insufficient	373 (11.92)	237 (11.49)	136 (12.75)	
Moderate	352 (11.25)	231 (11.20)	121 (11.34)	
High	1,628 (52.01)	1,135 (55.01)	493 (46.20)	
Survey cycle, n (%)				0.060
2007–2008	618 (19.74)	399 (19.34)	219 (20.52)	
2009–2010	685 (21.88)	428 (20.75)	257 (24.09)	
2011–2012	603 (19.27)	413 (20.02)	190 (17.81)	
2013–2014	651 (20.80)	450 (21.81)	201 (18.84)	
2015–2016	573 (18.31)	373 (18.08)	200 (18.74)	

IQR, interquartile range; PIR, poverty income ratio. *p* values were based on the Chi-square test (categorical variables) or the Wilcoxon rank sum test (continuous variables).

### Concentration distribution of PAH metabolites and their correlation

The characteristics of six PAH metabolites were shown in Supplementary Table S1. The detection rates of all six chemicals in urinary samples were greater than 80%. The median and IQR of concentrations (ng/ml) of 1-NAP, 2-NAP, 3-FLU, 2-FLU, 1-PHE, and 1-PYR among were 1602.75 (4773.00), 4518.50 (8360.00), 78.00 (176.00), 201.30 (388.00), 118.70 (151.00), 110.00 (171.00), respectively.

**Figure 2:**
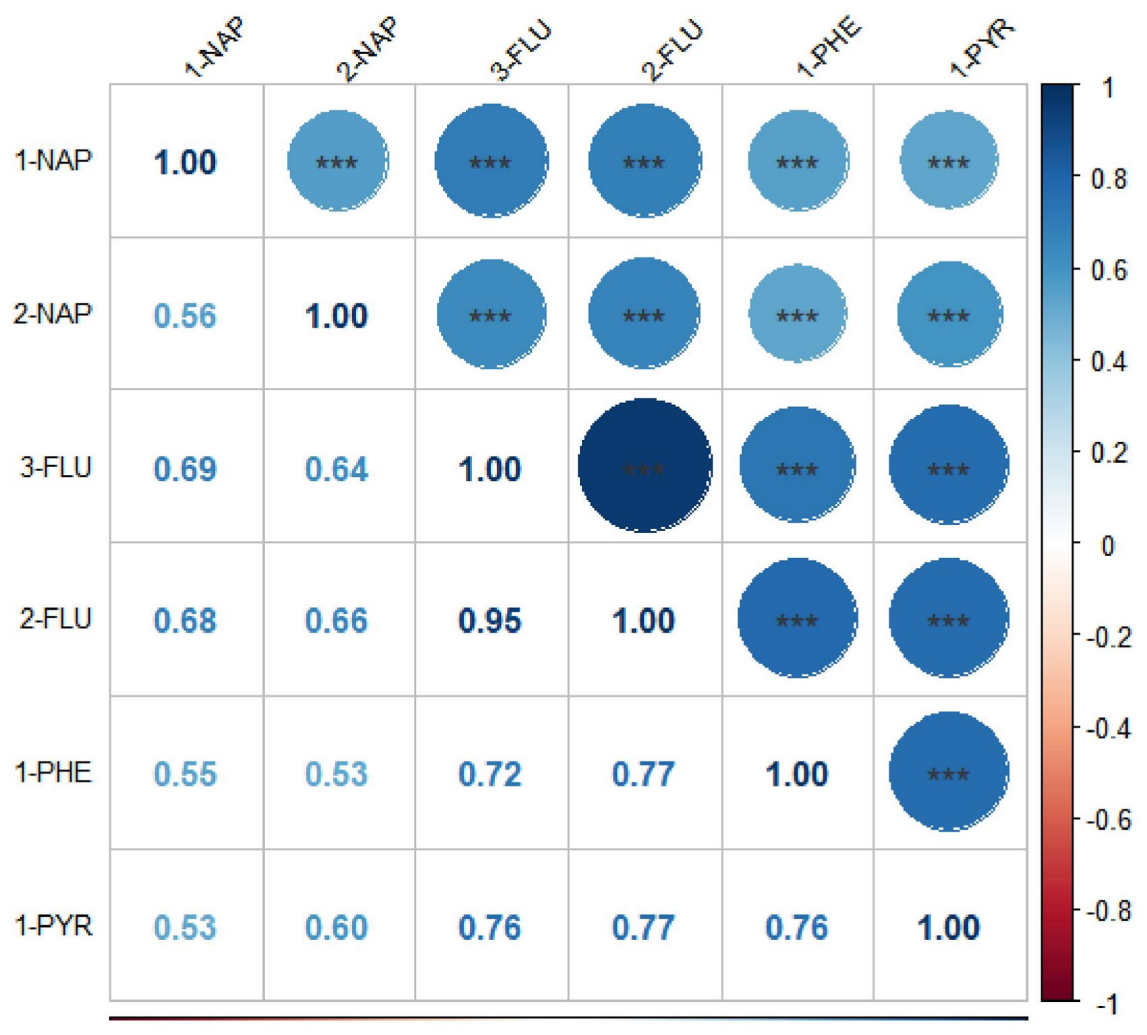
Spearman correlation coefficients among six PAH metabolites (National Health and Nutrition Examination Survey, United States, 2007–2016). All results for PAH metabolites were ln-transformed. *p < 0.05; **p < 0.01; ***p < 0.001.

The Spearman correlation coefficients between the ln-transformed PAH metabolites are depicted in Figure 2. All PAH metabolites were significantly and positively correlated with each other. 2-FLU and 3-FLU had the strongest correlation (*r* = 0.95, *p* < 0.001), followed by 2-FLU and 1-PHE (*r* = 0.77, *p* < 0.001), 2-FLU and 1-PYR (*r* = 0.77, *p* < 0.001).

### Association of PAHs with MASLD

To assess the individual impact of PAHs on MASLD, three multivariate Poisson regression models were constructed (Table 2). After adjusting all the covariates, in the continuous variable models, there were significant associations between 2-NAP and 1-PHE and MASLD risk with the *RR* values of 1.18 (95% *CI*: 1.08, 1.28) and 1.14 (1.04, 1.25) respectively. Compared with Q1, the higher quartiles of 2-NAP [Q2: 1.35 (1.06, 1.73); Q3: 1.67 (1.35, 2.07); Q4: 1.62 (1.23, 2.15); *p*-trend < 0.001], 2-FLU [Q3: 1.36 (1.08, 1.70); *p*-trend = 0.073], 1-PHE [Q4: 1.35 (1.03, 1.76); *p*-trend = 0.009], and 1-PYR [Q3: 1.37 (1.12, 1.69); Q4: 1.33 (1.01, 1.76); *p*-trend = 0.025] exhibited a heightened risk of MASLD in the fully adjusted model.

**Table 2:** Relative risks (95% confidence intervals) for the associations between PAHs and MASLD (National Health and Nutrition Examination Survey, United States, 2007–2016).

Variables		Model 1	Model 2	Model 3
		*RR* (95% *CI*)	*RR* (95% *CI*)	*RR* (95% *CI*)
1-NAP	Continuous	1.02 (0.92, 1.12)	1.07 (0.97, 1.18)	0.99 (0.94, 1.05)
	Q1	Ref	Ref	Ref
	Q2	0.98 (0.78, 1.23)	0.91 (0.72, 1.16)	0.91 (0.71, 1.15)
	Q3	1.01 (0.80, 1.27)	0.91 (0.72, 1.15)	0.92 (0.73, 1.16)
	Q4	0.93 (0.74, 1.17)	0.83 (0.66, 1.05)	0.84 (0.65, 1.08)
	*p*-trend	0.590	0.140	0.205
2-NAP	Continuous	1.14 (1.03, 1.25)*	1.12 (1.02, 1.23)*	1.18 (1.08, 1.28)**
	Q1	Ref	Ref	Ref
	Q2	1.32 (1.02, 1.71)*	1.36 (1.06, 1.74)*	1.35 (1.06, 1.73)*
	Q3	1.57 (1.26, 1.97)***	1.67 (1.34, 2.07)***	1.67 (1.35, 2.07)***
	Q4	1.48 (1.13, 1.94)**	1.56 (1.20, 2.04)***	1.62 (1.23, 2.15)***
	*p*-trend	0.001	<0.001	<0.001
3-FLU	Continuous	1.02 (0.94, 1.11)	1.03 (0.95, 1.11)	0.97 (0.89, 1.05)
	Q1	Ref	Ref	Ref
	Q2	0.87 (0.73, 1.05)	0.87 (0.73, 1.03)	0.86 (0.72, 1.03)
	Q3	0.93 (0.73, 1.18)	0.95 (0.77, 1.17)	0.97 (0.78, 1.21)
	Q4	0.85 (0.67, 1.08)	0.87 (0.69, 1.10)	0.90 (0.68, 1.19)
	*p*-trend	0.301	0.443	0.677
2-FLU	Continuous	0.95 (0.88, 1.02)	0.96 (0.90, 1.03)	1.06 (0.96, 1.17)
	Q1	Ref	Ref	Ref
	Q2	1.05 (0.84, 1.31)	1.07 (0.87, 1.32)	1.06 (0.86, 1.31)
	Q3	1.29 (1.01, 1.66)*	1.29 (1.03, 1.60)*	1.36 (1.08, 1.70)**
	Q4	1.16 (0.88, 1.51)	1.21 (0.93, 1.58)	1.30 (0.96, 1.78)
	*p*-trend	0.318	0.155	0.073
1-PHE	Continuous	1.13 (1.04, 1.22)**	1.15 (1.07, 1.24)***	1.14 (1.04, 1.25)***
	Q1	Ref	Ref	Ref
	Q2	0.94 (0.74, 1.18)	0.93 (0.73, 1.17)	0.93 (0.74, 1.18)
	Q3	1.25 (0.99, 1.58)	1.22 (0.97, 1.53)	1.25 (1.00, 1.58)
	Q4	1.32 (1.01, 1.74)*	1.29 (0.98, 1.70)	1.35 (1.03, 1.76)*
	*p*-trend	0.015	0.021	0.009
1-PYR	Continuous	1.01 (0.96, 1.06)	0.99 (0.93, 1.04)	1.09 (0.99, 1.21)
	Q1	Ref	Ref	Ref
	Q2	1.02 (0.83, 1.26)	1.09 (0.90, 1.33)	1.12 (0.93, 1.35)
	Q3	1.19 (0.96, 1.48)	1.33 (1.08, 1.64)**	1.37 (1.12, 1.69)**
	Q4	1.10 (0.84, 1.45)	1.26 (0.96, 1.66)	1.33 (1.01, 1.76)*
	*p*-trend	0.403	0.066	0.025

Poisson regression models were applied. All results for PAH metabolites were ln-transformed. Model 1: only adjusted for creatinine. Model 2: based on model 1 and adjusted for age, sex, race, educational level, marital status, PIR, and NHANES cycle. Model 3: based on model 2 and adjusted for cotinine and physical activity. Q1 to Q4 refer to the 1st through 4th quartiles of PAH metabolites. Ref, reference. *p < 0.05; **p < 0.01; ***p < 0.001.

### Subgroup analysis

We performed subgroup analysis by age to examine potential risk of MASLD (Supplementary Table S2). Significant interaction terms by age were observed for 1-NAP (*P*-interaction = 0.025) and 3-FLU (*P*-interaction = 0.027), and 2-FLU (*P*-interaction = 0.040). We found a positive relationship [*RR*s (95% *C*Is)] between 2-NAP [Q2: 1.92 (1.14, 3.23); Q3: 2.21 (1.31, 3.74); Q4: 2.22 (1.20, 4.11); *p*-trend = 0.012] and MASLD in the age group 20–39 years. Among people aged 40–59 years, there was an elevated risk of MASLD associated with the higher quartile exposure compared to Q1, including 2-NAP [Q3: 1.74 (1.18, 2.56); Q4: 1.65 (1.10, 2.46); *p*-trend = 0.007], 2-FLU [Q3: 1.79 (1.23, 2.60); Q4: 1.83 (1.23, 2.74); *p*-trend = 0.002]. In the continuous variable models, there were statistical associations between 2-NAP [1.21 (1.02, 1.44)] and 3-FLU [0.87 (0.77, 0.99)] and MASLD in the 20–39 age group. In the 40–59 age group, 2-NAP [1.19 (1.05, 1.35)], 2-FLU [1.18 (1.05, 1.32)], 1-PHE [1.26 (1.11, 1.42)], and 1-PYR [1.18 (1.01, 1.37)] were significant positively associated with MASLD. In individuals aged 60 years or older, only 2-NAP [1.12 (1.01, 1.24)] exposure was positively associated with MASLD risk.

Significant interaction term by sex was observed for 1-PYR (*P*-interaction = 0.042). In the female subgroup, individuals in the higher quartiles of 2-NAP [Q2: 1.54 (1.05, 2.26); Q3: 1.87 (1.31, 2.66); Q4: 1.95 (1.33, 2.86); *p*-trend < 0.001], 2-FLU [Q4: 1.76 (1.18, 2.63); *p*-trend = 0.007], 1-PHE [Q3: 1.52 (1.09, 2.12); Q4: 1.48 (1.01, 2.18); *p*-trend = 0.016], and 1-PYR [Q2: 1.36 (1.02, 1.80); Q3: 1.96 (1.41, 2.72); Q4: 1.91 (1.23, 2.95); *p*-trend = 0.002] were positively associated with MASLD (Supplementary Table S3). The results of continuous variable models are also consistent. In the male subgroup, 2-NAP [Q3: 1.51 (1.18, 1.93); Q4: 1.42 (1.02, 1.97); *p*-trend = 0.016] and 2-FLU [Q3: 1.48 (1.13, 1.94); *p*-trend = 0.417] were positively associated with MASLD. 2-NAP [1.13 (1.02, 1.24)] was associated with MASLD in the continuous variable models.

### Dose–response relationships between PAHs and MASLD

The RCS models represented the dose–response relationship between individual PAH metabolites and MASLD risk (Supplementary Figure S1). 2-NAP (*P*_for-overall_ < 0.01), 2-FLU (*P*_for-overall_ = 0.043), 1-PHE (*P*_for-overall_ = 0.002) and 1-PYR (*P*_for-overall_ = 0.008) were significantly associated with MASLD. No nonlinear dose–response relationship was found.

### Association of PAHs mixture exposure and MASLD

**Figure 3:**
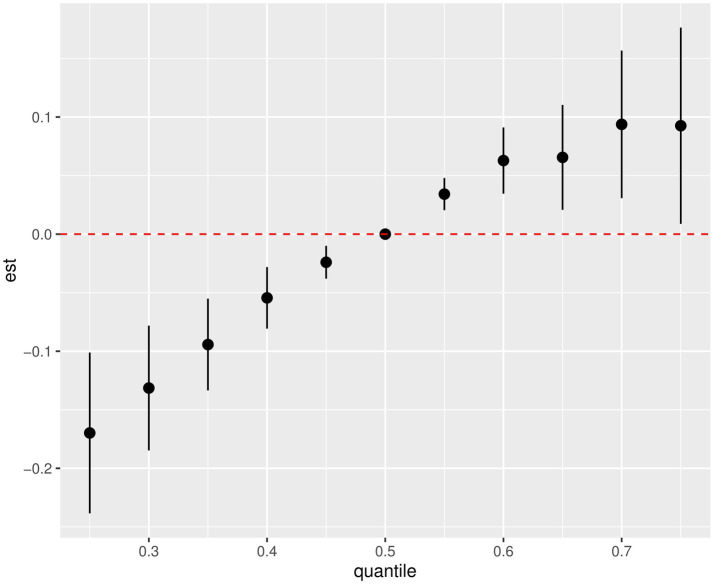
Combined effects of the PAHs mixture on MASLD risk estimated by BKMR (National Health and Nutrition Examination Survey, United States, 2007–2016). All results for PAH metabolites were ln-transformed. Covariates included creatinine age, sex, race, educational level, marital status, PIR, NHANES cycle, cotinine, and physical activity.

BKMR was performed to estimate the possible combined effect of the PAH metabolites mixture. The completely adjusted model exhibited a positive trend between mixed PAH metabolites and MASLD compared to the 50th percentiles (Figure 3). According to the posterior inclusion probability (PIP) of each PAH metabolite (Supplementary Table S4), 2-NAP and 3-FLU contributed the most to MASLD risk (PIP = 1), followed by 1-PHE (PIP = 0.9752). Additionally, by fixing the other exposure at its 25th, 50th or 75th percentile, we analyzed the individual effect of each exposure on the occurrence of MASLD (Supplementary Figure S2). Our results showed that 2-NAP, 2-FLU and 1-PHE were positively associated with increased risk of MASLD, and 3-FLU was inversely associated, when other metabolites were fixed at the 25th, 50th and 75th percentiles. Then, univariate exposure-response functions were assessed at fixed median concentrations of other PAHs (Supplementary Figure S3). Positive correlations were observed between 2-NAP, 2-FLU, 1-PHE, and the risk of MASLD, while 3-FLU showed the opposite relationship.

**Figure 4:**
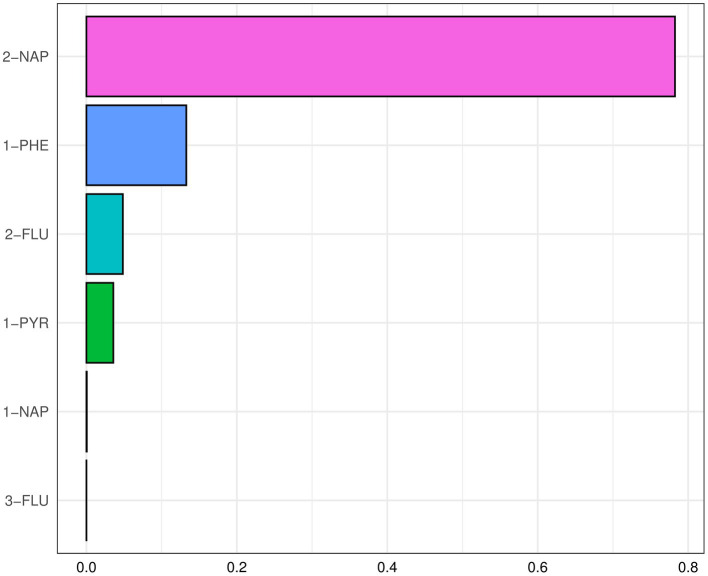
The WQS index weights of each of the six PAHs associated with MASLD (National Health and Nutrition Examination Survey, United States, 2007–2016). All results for PAH metabolites were ln-transformed. Covariates included creatinine age, sex, race, educational level, marital status, PIR, NHANES cycle, cotinine, and physical activity.

After adjusting for the covariate, a positive and significant association was observed between the WQS index for the six PAHs and MASLD [*OR* (95% *CI*): 1.25 (1.06, 1.49)] (Supplementary Table S5). 2-NAP and 1-PHE were identified as major PAHs, with weights of 78.24 and 13.28%, respectively (Figure 4). The WQS regression in the negative direction showed no significant association between PAHs mixture and MASLD [0.86 (0.75, 1.00)].

### Sensitivity analysis

Supplementary Tables S6–S8 indicated that even after considering HEI-2015 and occupational exposure as covariates, the association between PAHs and MASLD risk remains consistent (*p* < 0.05).

## Discussion

This study revealed that 2-NAP, 2-FLU, 1-PHE, and 1-PYR may be associated with an increased the risk of MASLD in American adults from the NHANES 2007–2016 dataset. The associations were significant those aged 20 to 59 years and in females. The RCS results showed that no nonlinear dose–response relationship was found. Moreover, the BKMR model revealed a significant and positive association between the PAH mixture and MASLD risk, with 2-NAP and 3-FLU identified as the main contributors. The WQS model indicated the PAHs mixture was positively associated with the risk of MASLD, primarily driven by 2-NAP and 1-PHE.

Although the exact mechanism of the link between PAHs exposure and MASLD is uncertain, there are several possible explanations. The liver is the primary organ responsible for metabolizing PAHs and contains high levels of CYP. The AhR is a ubiquitously expressed ligand-activated transcription factor with multiple physiological functions (33). Activation of the AhR by PAHs regulates the expression of CYP, including CYP1A1, CYP1A2 and CYP1B1 (16), with CYP1A1 serving as a typical marker of AhR activation (34). Overexpression of CYP1A1 can disrupt the estrogen signaling pathway and diminish the ability of 17β-estradiol to protect against hepatic steatosis, which is marked by the buildup of TG (17), a pathological feature of MASLD (35). Then, CYP1A1 is a key enzyme of oxidative stress, and its overexpression can cause oxidative stress by affecting reactive oxygen species (ROS) and superoxide dismutase (SOD) levels (18). An animal study also showed that markers of antioxidants such as glutathione-S-transferase, SOD and catalase were reduced in African catfish exposed to benzo[b]fluoranthene (36). Oxidative stress is known to play an important role in the pathogenesis of MASLD (37). Moreover, AhR is involved in the modulation of tumor necrosis factor alpha (TNF-*α*) and interleukin-8 (IL-8) expression (19). TNF-α interferes with insulin signaling promoting insulin resistance (20). Increased ROS levels are also an important trigger for insulin resistance (38). Insulin resistance may contribute significantly to the development of MASLD and its induced abnormalities in lipid metabolism can lead to increased production of proinflammatory cytokines, such as TNF-α, IL-1b, and IL-6, as well as less adiponectin, thereby inducing systemic insulin resistance (39). Low-grade chronic inflammation and systemic insulin resistance are crucial for mediating hepatic and most extrahepatic complications of MASLD (7).

Beside activation of AhR and CYP1A1, recent studies have shown that several PAHs, including fluoranthene, phenanthrene, and pyrene, interact with human and/or mouse constitutive androstane receptor (CAR), inducing its some target gene, such as CYP2B1, CYP2B2, CYP2B6, CYP2B10 (40, 41). CAR, highly expressed in the liver, belongs to the nuclear receptor superfamily. Animal experiments revealed that administration of pyrene or phenanthrene led to increased relative liver weight, hepatocellular hypertrophy, and higher serum ALT levels in wild-type mice, but these effects were absent in CAR-null mice (40, 41). Additionally, hepatic total glutathione (GSH) levels were lower in wild-type mice compared to CAR-null mice, indicating that GSH reduction may contribute to hepatotoxicity caused by pyrene or phenanthrene. In addition, another study in mice found that CAR is crucial in the liver inflammatory triggered by pyrene, marked by increased levels of serum amyloid A proteins and IL-17-producing helper T cells (42).

The evidence concerning the link between PAH exposure and the development of MASLD in the general population is inconclusive. Previous studies can provide some clues that PAHs exposure may cause NAFLD. A study from KoNEHS revealed that 2-hydroxyfluorene levels contributed the most to significantly increasing the prevalence of NAFLD using the BKMR model (25). Two studies utilizing NHANES data also identified a notable correlation between PAH mixtures and NAFLD risk (26, 43). These are consistent with our findings on MASLD. In addition, exposure to PAHs is linked to abnormal liver function indices. Previous studies suggested that 2-NAP and 2-FLU were linked to the increase in ALT, AST, and GGT (23). Serum bilirubin, also one of the liver blood test indicators, has antioxidant and cytoprotective effects, and its level is inversely correlated with NAFLD (44). A study of American adults found negative associations between 2-NAP and 1-PYR and total bilirubin (45). This study found that 3-FLU may be associated with MASLD. In the BKMR model, 3-FLU exhibited a negative association with MASLD risk, which is consistent with the direction of association observed in subgroup analyses, and was one of the primary contributors in mixed exposure to pollutants. The single-pollutant regression showed a negative correlation between 3-FLU and MASLD, but without statistical significance. Compared to single-pollutant models, BKMR overcomes the disadvantages of traditional methods that may be limited by multicollinearity and model selection errors. BKMR allows for simultaneous variable selection and effect estimation, capturing nonlinear and non-additive effects among co-exposures, and thus better capturing the combined effect of mixtures and individual effect of single chemical in the mixture, such as the relationship between 3-FLU and MASLD (30, 46). Although the specific mechanism is unclear, previous studies have found that 3-FLU was negatively correlated with other adverse outcomes. Studies found significant negative associations between 3-FLU and NAFLD and MAFLD, which researchers propose is consistent with previous findings on synergistic or antagonistic effects existed in PAHs (26, 47). Research on PAHs and osteoporosis showed that 2-FLU was associated with an increased prevalence of osteoporosis, whereas 3-FLU was associated with a decreased prevalence of osteoporosis (48). Another study among U.S. adults found an inverse association between 3-FLU exposure and cervical cancer prevalence, and speculated that this association may be related to the methylation levels of the AhR repressor gene (49, 50). Further research is needed to better understand the underlying mechanisms involved in these associations.

The definition of NAFLD mainly focuses on excluding other factors responsible for hepatic steatosis. While MASLD also includes patients with obesity, insulin resistance, vascular dysfunction, or dyslipidemia (51). Chronic exposure to low-molecular-weight PAHs, like 2-NAP, results in lipid buildup in adipocytes and triggers inflammation, demonstrating an obesogenic potential (52). A systematic review with meta-analysis found that naphthalene, phenanthrene, and total OH-PAHs metabolites were significantly positively correlated with the risk of obesity (53). A study on *Xenopus tropicalis* reported hepatotoxicity caused by impairment of lipid and cholesterol metabolism in individuals exposed to benzo[a]pyrene (B[a]p) (54). A study in China showed that the sum concentrations of hydroxyphenanthrene were positively associated with an average increase of serum concentrations of total cholesterol or low-density lipoprotein-cholesterol over 6 years (55). Moreover, phenanthrene can interfere with adipocytokine levels through epigenetic modification, which in turn affects glucose metabolism, leading to insulin resistance (56). In a study of a nationally representative sample of U.S. adults, PAHs exposure was found to be associated with increased insulin resistance, impaired *β* cell function, and an increased prevalence of metabolic syndrome (21). A study involving Korean women found that PAHs may contribute to the pathogenesis of insulin resistance through methylation mediated inhibition of the IRS2 gene (22). Additionally, a study performed both *in vivo* and *in vitro* proved that chronic exposure to PAHs can induce endothelial dysfunction in rats and primary human umbilical vein endothelial cells, resulting in cardiometabolic disease (57). A recent systematic review found a positive association between PAHs and CVD (57). A study using NHANES data revealed that joint exposure to PAHs showed positive association with CVD and all-cause mortality (58). Thus, PAHs exposure may contribute to the development of MASLD. However, strong evidence is still needed to further prove the relationship between PAHs and MASLD.

Furthermore, the subgroup analysis results suggest that there are sex differences in the association between exposure to PAHs and MASLD. A European flounder experiment proposed that after exposure to (B[a]p), female flounder hepatocytes exhibited slower biotransformation and lower capacity for non-enzymatic antioxidant defense and detoxification of toxic aldehydes than males, which may indicate that females are more sensitive to environmental toxicants and carcinogens (59). There are also differences between different age groups. Young and middle-aged people have chronic exposure to elevated levels of PAHs because of their occupations (60), such as coke oven workers (61) and firefighters (62). In addition, they may be more likely to be exposed to tobacco. And tobacco users had higher PAH urinary biomarker levels compared to non-users (63). Moreover, Estrogen has a potential protective effect in alleviating the onset and progression of MASLD (64). The current median age of menopause is 52 years in the United States, ranging from 45 to 55 years (65). In the age group of 40–59, compared to younger groups, women may experience menopause, with a significant decrease in estrogen levels, thereby increasing the risk of MASLD.

## Strengths and limitations

This study exhibits several notable advantages. This is an inaugural investigation into the relationship between PAHs exposure and MASLD in a nationally representative sample. Then the large and nationally representative nature of the sample in this study boosts its statistical power, allowing for robust detection of associations. Additionally, the effect of PAHs on MASLD was verified using a variety of methods, and the consistency of results across these methods lends further support to the conclusions drawn. The limitations of our study should be recognized. Firstly, it is not possible to confirm a causal relationship between PAHs and MASLD, given the cross-sectional nature of the study. To further confirm our findings, it would be imperative to conduct cohort designs and animal studies. Secondly, urine PAHs samples were collected and measured from participants at a single time point, so continuous exposure and intra-individual differences may not be reflected. Moreover, despite the proven utility and ethicality of this method in numerous epidemiological studies, the current gold standard for diagnosing fatty liver remains still invasive liver biopsy. Additionally, while viral hepatitis (HBV/HCV) and significant alcohol use were excluded according to MASLD criteria, NHANES lacks data to systematically rule out other competing etiologies of hepatic steatosis (e.g., drug-induced liver injury, autoimmune hepatitis, or hereditary hemochromatosis) as strictly required by the Delphi consensus, which indicates that some cases classified as MASLD may have coexisting or alternative underlying causes. Future studies incorporating specialized laboratory tests and medication histories could help refine the diagnostic accuracy. Lastly, despite controlling for many confounding factors, residual unknown confounding factors may still exist, which could influence our results. Our study belongs to exploratory research, the purpose of which is to preliminarily explore the relationship between PAHs and MASLD, so we did not perform adjustment for the level of significance.

## Conclusion

The results of our study suggested that individual or combined exposure to PAHs may be associated with an increased risk of MASLD in American adults. These associations were not identical between males and females, and there were also differences across age groups. Exploring the independent and/or combined effects of environmental factors on MASLD is crucial for disease prevention. Protecting key populations and reducing occupational exposures can be effective in preventing the harms of PAHs. However, further longitudinal studies and biological mechanism studies are needed to establish causality.

## Data Availability

Publicly available datasets were analyzed in this study. This data can be found at: https://www.cdc.gov/nchs/nhanes/index.htm.

## Footnotes

^1^ https://www.cdc.gov/nchs/nhanes/index.htm
