# Supplementary material for: Association between polycyclic aromatic hydrocarbons exposure and metabolic dysfunction-associated steatotic liver disease in US adults
Source: Front Public Health. 2025 Jun 11;13:1540357. doi: 10.3389/fpubh.2025.1540357 (PMC12187730; doi:10.3389/fpubh.2025.1540357)
Supplement: Supplementary file 1 [file Data_Sheet_1.docx]

**Supplemental Materials**

**Supplementary Methods**

**Table S1** Concentrations and detection frequencies of urinary PAH metabolites (ng/L) in NHANES 2007-2016.

**Table S2** Association of PAHs with MASLD after age subgroup in NHANES 2007–2016.

**Table S3** Association of PAHs with MASLD after sex subgroup in NHANES 2007–2016.

**Table S4** PIPs of each PAH in BKMR model.

**Table S5** Estimation of the combined effect of PAHs on the risk of MASLD using WQS.

**Table S6.** Relative risks (95% confidence intervals) for the associations between PAHs and MASLD (National Health and Nutrition Examination Survey, The United States, 2007–2016)

**Table S7.** Relative risks (95% confidence intervals) for the associations between PAHs and MASLD (National Health and Nutrition Examination Survey, The United States, 2007–2012)

**Table S8.** Relative risks (95% confidence intervals) for the associations between PAHs and MASLD (National Health and Nutrition Examination Survey, The United States, 2007–2012)

**Fig. S1** The results of restricted cubic spline (RCS) model of MASLD and individual PAH metabolites in full adjusted model.

**Fig. S2** Associations of single PAHs with MASLD risk were estimated by BKMR models, when other all PAHs were held at their corresponding 25th (red), 50th (green), or 75th (blue) percentile, respectively.

**Fig. S3** Univariate exposure-response functions (95%CI) for single PAHs when other PAHs fixed at the median.

**Supplementary Methods**

**1.Measurement of PAH metabolites**

In the NHANES 2007-2008 cycle, the analysis was performed by capillary gas chromatography combined with high resolution mass spectrometry (GC/HRMS) with limits of detection (LOD) of 48 pg mL−1 for 1- NAP, 13 pg mL−1 for 2-NAP, and 5 pg mL−1 for others. In the NHANES 2009-2012 cycle, the analysis was performed by isotope dilution capillary gas chromatography tandem mass spectrometry (GC-MS/MS) with LODs of 44 pg mL−1 for 1-NAP, 42 pg mL−1 for 2-NAP, and 10 pg mL−1 for others. In the NHANES 2013-2016 cycle, the analysis was performed by isotope dilution high performance liquid chromatography-tandem mass spectrometry (on-line SPE-HPLC- MS/MS) with LODs of 60 pg mL−1 for 1-NAP, 90 pg mL−1 for 2-NAP, 8 pg mL−1 for 2-FLU and 3-FLU, 9 pg mL−1 for 1-PHE, and 70 pg mL−1 for 1-PYR. If the value was below the LOD, it was substituted with the LOD divided by the square root of 2. The detailed introduction can be found at the following website link:

https://wwwn.cdc.gov/Nchs/Nhanes/2007-2008/PAH_E.htm#URXUCR; https://wwwn.cdc.gov/Nchs/Nhanes/2009-2010/PAH_F.htm; https://wwwn.cdc.gov/Nchs/Nhanes/2013-2014/PAH_H.htm.

**2.The details of covariates**

Sex is categorised as male or female. Race/ethnicity were recorded according to the categories "Non-Hispanic White," "Non-Hispanic Black", "Mexican American", and "Other races" that included "Other Hispanic" and the category of "other race". Educational levels were grouped into five categories, including less than high school, high school graduate/the General Education Development test or equivalent, and college or above. Marital status was classified as married/cohabiting, never, and widowed/divorced/separated. The poverty income ratio (PIR) was dichotomized into two categories: PIR <1, and PIR ≥1. The measurement of physical activity was performed in terms of metabolic equivalents (MET)(Fowler et al. 2020). Physical activity was grouped into four levels: sedentary (no physical activity), insufficient (<500 MET-minutes per week), moderate (≥500 and <1000 MET-minutes per week) and high (≥1000 MET-minutes per week). The mean number of alcoholic beverages consumed daily was calculated by multiplying the number of days on which alcohol was consumed over the past 12 months by the mean number of drinks consumed per drinking day and dividing the result by 365 days.(Lazo et al. 2011). According to U.S. standards, each alcoholic drink was considered to contain 14 grams of alcohol(van Kleef et al. 2023). Excessive alcohol consumption was defined as a daily intake of ≥20 grams for females and ≥30 grams for males. Cotinine concentration was used to estimate individual exposure to tobacco. Urinary creatinine is included as a continuous variable in the covariates to balance the levels of urinary metabolites. T2DM was defined as HbA1c ≥6.5%, or FPG ≥7.0 mmol/L, or 2hPG ≥11.1 mmol/L, or self-report of a diagnosis by a physician or health care professional, or self-reported use of diabetic pills.

The Healthy Eating Index (HEI) -2015 is an indicator for evaluating dietary quality, aimed at aligning with the 2015-2020 Dietary Guidelines for Americans. The HEI-2015 ingredient list is the same as in HEI-2010, except that saturated fat and added sugars replace empty calories, resulting in 13 ingredients. HEI-2015 scores range from 0-100, with higher HEI scores reflecting better diet quality(Krebs-Smith et al. 2018). NHANES participants were asked four questions about workplace exposure to specific substances. Participants were considered to have occupational exposure if they answered "yes" to any of the following questions regarding workplace contact with mineral dust ("from rock, sand, concrete, coal, asbestos, silica or soil"), organic dust ("from baking flours, grains, wood, cotton, plants or animals"), exhaust fumes ("from trucks, buses, heavy machinery or diesel engines"), or other gases, vapors, or smoke ("vapors from paints, cleaning products, glues, solvents, and acids; or welding/soldering fumes")(Doney et al. 2019).

**Reference**

Doney B, Kurth L, Halldin C, Hale J, Frenk SM (2019) Occupational exposure and airflow obstruction and self-reported COPD among ever-employed US adults using a COPD-job exposure matrix Am J Ind Med 62:393-403 doi:10.1002/ajim.22958

Fowler JR, Tucker LA, Bailey BW, LeCheminant JD (2020) Physical Activity and Insulin Resistance in 6,500 NHANES Adults: The Role of Abdominal Obesity J Obes 2020:3848256 doi:10.1155/2020/3848256

Krebs-Smith SM et al. (2018) Update of the Healthy Eating Index: HEI-2015 J Acad Nutr Diet 118:1591-1602 doi:10.1016/j.jand.2018.05.021

Lazo M, Hernaez R, Bonekamp S, Kamel IR, Brancati FL, Guallar E, Clark JM (2011) Non-alcoholic fatty liver disease and mortality among US adults: prospective cohort study BMJ 343:d6891 doi:10.1136/bmj.d6891

van Kleef LA, de Knegt RJ, Brouwer WP (2023) Metabolic dysfunction-associated fatty liver disease and excessive alcohol consumption are both independent risk factors for mortality Hepatology 77:942-948 doi:10.1002/hep.32642

**Table S1** Concentrations and detection frequencies of urinary PAH metabolites (ng/L) in NHANES 2007-2016.

| Variables (ng/L) | DF (%) | GM | Percentile | | | | |
| --- | --- | --- | --- | --- | --- | --- | --- |
|  |  |  | 5th | 25th | 50th | 75th | 95th |
| 1-NAP | 99.94 | 2036.538 | 258 | 698 | 1602.75 | 5471 | 25800 |
| 2-NAP | 100 | 4544.029 | 716.1 | 2004 | 4518.5 | 10364 | 29730.9 |
| 3-FLU | 99.07 | 98.769 | 16 | 38 | 78 | 214 | 1278 |
| 2-FLU | 100 | 234.8458 | 41 | 102 | 201.3 | 490 | 2212 |
| 1-PHE | 99.84 | 118.7585 | 28 | 64 | 118.7 | 215 | 521.6 |
| 1-PYR | 88.05 | 116.5701 | 27.5 | 49.5 | 110 | 220.5 | 615 |

Note: DF, detection frequency; GM, geometric mean.

**Table S2** Association of PAHs with MASLD after age subgroup in NHANES 2007–2016.

| Chemicals |  | 20-39 | |  | 40-59 | |  | ≥60 | |  |
| --- | --- | --- | --- | --- | --- | --- | --- | --- | --- | --- |
|  |  | RR (95% CI) | *P*-trend |  | RR (95% CI) | *P*-trend |  | RR (95% CI) | *P*-trend | *P ^a^* |
| 1-NAP | Continuous | 0.87 (0.76, 1.00) |  |  | 1.05 (0.98, 1.12) |  |  | 1.00 (0.92, 1.08) |  | 0.025 |
|  | Q1 | Reference | 0.045 |  | Reference | 0.729 |  | Reference | 0.482 |  |
|  | Q2 | 1.05 (0.71, 1.57) |  |  | 0.82 (0.56, 1.18) |  |  | 0.86 (0.62, 1.20) |  |  |
|  | Q3 | 0.83 (0.54, 1.28) |  |  | 0.94 (0.66, 1.32) |  |  | 0.98 (0.73, 1.31) |  |  |
|  | Q4 | 0.62 (0.37, 1.04) |  |  | 0.99 (0.71, 1.39) |  |  | 0.85 (0.60, 1.19) |  |  |
| 2-NAP | Continuous | **1.21 (1.02, 1.44)*** |  |  | **1.19 (1.05, 1.35)**** |  |  | **1.12 (1.01, 1.24)*** |  | 0.565 |
|  | Q1 | Reference | 0.012 |  | Reference | 0.007 |  | Reference | 0.045 |  |
|  | Q2 | **1.92 (1.14, 3.23)*** |  |  | 1.35 (0.86, 2.12) |  |  | 1.12 (0.83, 1.51) |  |  |
|  | Q3 | **2.21 (1.31, 3.74)**** |  |  | **1.74 (1.18, 2.56)**** |  |  | 1.30 (0.97, 1.75) |  |  |
|  | Q4 | **2.22 (1.20, 4.11)*** |  |  | **1.65 (1.10, 2.46)*** |  |  | 1.36 (0.97, 1.89) |  |  |
| 3-FLU | Continuous | **0.87 (0.77, 0.99)*** |  |  | 1.06 (0.95, 1.19) |  |  | 0.90 (0.81, 1.01) |  | 0.027 |
|  | Q1 | Reference | 0.080 |  | Reference | 0.107 |  | Reference | 0.113 |  |
|  | Q2 | 0.89 (0.59, 1.35) |  |  | 0.92 (0.67, 1.27) |  |  | 0.76 (0.59, 0.99) |  |  |
|  | Q3 | 0.83 (0.54, 1.28) |  |  | 1.26 (0.90, 1.75) |  |  | **0.67 (0.50, 0.91)*** |  |  |
|  | Q4 | 0.65 (0.39, 1.09) |  |  | 1.27 (0.87, 1.86) |  |  | 0.76 (0.55, 1.05) |  |  |
| 2-FLU | Continuous | 1.02 (0.86, 1.20) |  |  | **1.18 (1.05, 1.32)**** |  |  | 0.94 (0.82, 1.07) |  | 0.040 |
|  | Q1 | Reference | 0.567 |  | Reference | 0.002 |  | Reference | 0.409 |  |
|  | Q2 | 1.38 (0.86, 2.22) |  |  | 1.07 (0.75, 1.54) |  |  | 0.86 (0.64, 1.14) |  |  |
|  | Q3 | 1.35 (0.88, 2.06) |  |  | **1.79 (1.23, 2.60)**** |  |  | 0.98 (0.72, 1.33) |  |  |
|  | Q4 | 1.28 (0.75, 2.20) |  |  | **1.83 (1.23, 2.74)**** |  |  | 0.82 (0.55, 1.22) |  |  |
| 1-PHE | Continuous | 1.09 (0.92, 1.30) |  |  | **1.26 (1.11, 1.42)***** |  |  | 1.02 (0.87, 1.20) |  | 0.097 |
|  | Q1 | Reference | 0.313 |  | Reference | 0.022 |  | Reference | 0.119 |  |
|  | Q2 | 0.95 (0.59, 1.54) |  |  | 0.97 (0.64, 1.45) |  |  | 0.85 (0.60, 1.21) |  |  |
|  | Q3 | 1.46 (0.96, 2.21) |  |  | 1.10 (0.78, 1.56) |  |  | 1.10 (0.80, 1.51) |  |  |
|  | Q4 | 1.20 (0.70, 2.08) |  |  | 1.46 (0.99, 2.15) |  |  | 1.26 (0.90, 1.75) |  |  |
| 1-PYR | Continuous | 1.00 (0.84, 1.18) |  |  | **1.18 (1.01, 1.37)*** |  |  | 1.01 (0.88, 1.17) |  | 0.146 |
|  | Q1 | Reference | 0.252 |  | Reference | 0.081 |  | Reference | 0.569 |  |
|  | Q2 | 0.84 (0.49, 1.45) |  |  | 1.25 (0.88, 1.78) |  |  | 1.14 (0.90, 1.46) |  |  |
|  | Q3 | 1.38 (0.89, 2.13) |  |  | 1.37 (0.98, 1.93) |  |  | 1.27 (0.97, 1.67) |  |  |
|  | Q4 | 1.22 (0.72, 2.08) |  |  | 1.51 (0.95, 2.39) |  |  | 1.04 (0.72, 1.51) |  |  |

Note: All results for PAH metabolites were ln-transformed. Models were adjusted for creatinine, sex, race, educational attainment, marital status, PIR, NHANES cycle, cotinine, and physical activity. Q1 to Q4 refer to the 1st through 4th quartiles of PAH metabolites. **P* < 0.05; ***P* < 0.01; ****P* < 0.001.

^a^: *P*-interaction

**Table S3** Association of PAHs with MASLD after sex subgroup in NHANES 2007–2016.

| Chemicals |  | Male | |  | Female | |  |
| --- | --- | --- | --- | --- | --- | --- | --- |
|  |  | RR (95% CI) | *P*-trend |  | RR (95% CI) | *P*-trend | *P ^a^* |
| 1-NAP | Continuous | 0.97 (0.90, 1.03) |  |  | 1.02 (0.94, 1.11) |  | 0.132 |
|  | Q1 | Reference | 0.228 |  | Reference | 0.310 |  |
|  | Q2 | 1.01 (0.75, 1.35) |  |  | 0.75 (0.53, 1.07) |  |  |
|  | Q3 | 0.92 (0.69, 1.23) |  |  | 0.92 (0.69, 1.23) |  |  |
|  | Q4 | 0.87 (0.65, 1.17) |  |  | 0.80 (0.58, 1.09) |  |  |
| 2-NAP | Continuous | **1.13 (1.02, 1.24)*** |  |  | **1.24 (1.11, 1.39)***** |  | 0.096 |
|  | Q1 | Reference | 0.016 |  | Reference | <0.001 |  |
|  | Q2 | 1.21 (0.90, 1.62) |  |  | **1.54 (1.05, 2.26)*** |  |  |
|  | Q3 | **1.51 (1.18, 1.93)**** |  |  | **1.87 (1.31, 2.66)**** |  |  |
|  | Q4 | **1.42 (1.02, 1.97)*** |  |  | **1.95 (1.33, 2.86)**** |  |  |
| 3-FLU | Continuous | 0.96 (0.87, 1.06) |  |  | 0.98 (0.86, 1.13) |  | 0.341 |
|  | Q1 | Reference | 0.293 |  | Reference | 0.435 |  |
|  | Q2 | 0.95 (0.71, 1.26) |  |  | **0.77 (0.60, 0.99)*** |  |  |
|  | Q3 | 1.09 (0.81, 1.47) |  |  | 0.80 (0.57, 1.13) |  |  |
|  | Q4 | 0.84 (0.60, 1.18) |  |  | 1.20 (0.80, 1.80) |  |  |
| 2-FLU | Continuous | 1.02 (0.91, 1.14) |  |  | **1.17 (1.00, 1.36)*** |  | 0.184 |
|  | Q1 | Reference | 0.417 |  | Reference | 0.007 |  |
|  | Q2 | 1.06 (0.78, 1.44) |  |  | 1.04 (0.78, 1.39) |  |  |
|  | Q3 | **1.48 (1.13, 1.94)**** |  |  | 1.15 (0.81, 1.62) |  |  |
|  | Q4 | 1.18 (0.83, 1.68) |  |  | **1.76 (1.18, 2.63)**** |  |  |
| 1-PHE | Continuous | 1.12 (1.00, 1.27) |  |  | **1.16 (1.01, 1.33)*** |  | 0.781 |
|  | Q1 | Reference | 0.139 |  | Reference | 0.016 |  |
|  | Q2 | 0.87 (0.64, 1.18) |  |  | 0.97 (0.69, 1.36) |  |  |
|  | Q3 | 1.04 (0.77, 1.39) |  |  | **1.52 (1.09, 2.12)*** |  |  |
|  | Q4 | 1.20 (0.85, 1.67) |  |  | **1.48 (1.01, 2.18)*** |  |  |
| 1-PYR | Continuous | 1.03 (0.91, 1.16) |  |  | **1.25 (1.07, 1.46)**** |  | 0.042 |
|  | Q1 | Reference | 0.538 |  | Reference | 0.002 |  |
|  | Q2 | 0.95 (0.72, 1.26) |  |  | **1.36 (1.02, 1.80)*** |  |  |
|  | Q3 | 1.05 (0.80, 1.37) |  |  | **1.96 (1.41, 2.72)***** |  |  |
|  | Q4 | 1.07 (0.78, 1.47) |  |  | **1.91 (1.23, 2.95)**** |  |  |

Note: All results for PAH metabolites were ln-transformed. Models were adjusted for creatinine, age, race, educational attainment, marital status, PIR, NHANES cycle, cotinine, and physical activity. Q1 to Q4 refer to the 1st through 4th quartiles of PAH metabolites. **P* < 0.05; ***P* < 0.01; ****P* < 0.001.

^a^: *P*-interaction

**Table S4** PIPs of each PAH in BKMR model.

| Variables | PIPs |
| --- | --- |
| 1-NAP | 0.9312 |
| 2-NAP | 1 |
| 3-FLU | 1 |
| 2-FLU | 0.9624 |
| 1-PHE | 0.9752 |
| 1-PYR | 0.3968 |

Note: This model adjusted for all covariates. PIP, posterior inclusion probability; BKMR, Bayesian kernel machine regression.

**Table S5** Estimation of the combined effect of PAHs on the risk of MASLD using WQS.

| Related Directions | *OR* (95% *CI*) | *P*-value | Component | Weight (%) |
| --- | --- | --- | --- | --- |
| Positive | 1.25 (1.06, 1.49) | 0.009 | 2-NAP | 78.24 |
|  |  |  | 1-PHE | 13.28 |
|  |  |  | 2-FLU | 4.85 |
|  |  |  | 1-PYR | 3.58 |
|  |  |  | 1-NAP | 0.05 |
|  |  |  | 3-FLU | 0.00 |
| Negative | 0.86 (0.75, 1.00) | 0.051 | 3-FLU | 54.75 |
|  |  |  | 1-NAP | 41.69 |
|  |  |  | 1-PYR | 2.80 |
|  |  |  | 1-PHE | 0.58 |
|  |  |  | 2-FLU | 0.19 |
|  |  |  | 2-NAP | 0.00 |

Note: All results for PAH metabolites were ln-transformed. Covariates included creatinine, age, sex, race, educational level, marital status, PIR, NHANES cycle, cotinine, and physical activity. Weight (%) shows individual contributions to the WQS index. WQS: Weighted Quantile Sum.

**Table S6.** Relative risks (95% confidence intervals) for the associations between PAHs and MASLD (National Health and Nutrition Examination Survey, The United States, 2007–2016)

| Variables |  | *RR* (95% *CI*) |
| --- | --- | --- |
| 1-NAP | Continuous | 0.99 (0.93, 1.05) |
|  | Q1 | Ref |
|  | Q2 | 0.95 (0.75, 1.21) |
|  | Q3 | 1.00 (0.79, 1.28) |
|  | Q4 | 0.88 (0.67, 1.16) |
|  | *P*-trend | 0.422 |
| 2-NAP | Continuous | 1.15 (1.05, 1.26)** |
|  | Q1 | Ref |
|  | Q2 | 1.32 (1.00, 1.73)* |
|  | Q3 | 1.68 (1.33, 2.13)*** |
|  | Q4 | 1.49 (1.10, 2.02)* |
|  | *P*-trend | 0.002 |
| 3-FLU | Continuous | 0.95 (0.87, 1.04) |
|  | Q1 | Ref |
|  | Q2 | 0.83 (0.66, 1.03) |
|  | Q3 | 0.94 (0.73, 1.21) |
|  | Q4 | 0.89 (0.67, 1.20) |
|  | *P*-trend | 0.713 |
| 2-FLU | Continuous | 1.04 (0.94, 1.16) |
|  | Q1 | Ref |
|  | Q2 | 0.98 (0.78, 1.24) |
|  | Q3 | 1.34 (1.03, 1.76)* |
|  | Q4 | 1.21 (0.87, 1.70) |
|  | *P*-trend | 0.164 |
| 1-PHE | Continuous | 1.11(1.01,1.22)* |
|  | Q1 | Ref |
|  | Q2 | 0.96 (0.75, 1.24) |
|  | Q3 | 1.19 (0.93, 1.53) |
|  | Q4 | 1.26 (0.92, 1.72) |
|  | *P*-trend | 0.076 |
| 1-PYR | Continuous | 1.06 (0.96, 1.18) |
|  | Q1 | Ref |
|  | Q2 | 1.08 (0.88, 1.32) |
|  | Q3 | 1.32 (1.06, 1.66)* |
|  | Q4 | 1.24 (0.95, 1.64) |
|  | *P*-trend | 0.071 |

Note: Poisson regression models were applied. All results for PAH metabolites were ln-transformed. Model adjusted for creatinine, age, sex, race, educational level, marital status, PIR, NHANES cycle, cotinine, physical activity, and HEI-2015. Q1 to Q4 refer to the 1st through 4th quartiles of PAH metabolites. Ref, reference. **P* < 0.05; ***P* < 0.01; ****P* < 0.001.

**Table S7.** Relative risks (95% confidence intervals) for the associations between PAHs and MASLD (National Health and Nutrition Examination Survey, The United States, 2007–2012)

| Variables |  | *RR* (95% *CI*) |
| --- | --- | --- |
| 1-NAP | Continuous | 1.02 (0.95, 1.10) |
|  | Q1 | Ref |
|  | Q2 | 0.96 (0.73, 1.27) |
|  | Q3 | 0.89 (0.70, 1.13) |
|  | Q4 | 0.87 (0.66, 1.16) |
|  | *P*-trend | 0.278 |
| 2-NAP | Continuous | 1.22 (1.09, 1.36)*** |
|  | Q1 | Ref |
|  | Q2 | 1.37 (0.95, 1.97) |
|  | Q3 | 1.86 (1.42, 2.44)*** |
|  | Q4 | 1.71 (1.19, 2.46)** |
|  | *P*-trend | <0.001 |
| 3-FLU | Continuous | 0.99 (0.89, 1.11) |
|  | Q1 | Ref |
|  | Q2 | 0.93 (0.71, 1.23) |
|  | Q3 | 1.11 (0.80,1.55) |
|  | Q4 | 1.09 (0.74, 1.61) |
|  | *P*-trend | 0.447 |
| 2-FLU | Continuous | 1.12 (0.99, 1.27) |
|  | Q1 | Ref |
|  | Q2 | 1.13 (0.84, 1.51) |
|  | Q3 | 1.64 (1.14, 2.36)** |
|  | Q4 | 1.61 (1.08, 2.39)* |
|  | *P*-trend | 0.012 |
| 1-PHE | Continuous | 1.17 (1.07, 1.28)*** |
|  | Q1 | Ref |
|  | Q2 | 1.24 (0.92, 1.67) |
|  | Q3 | 1.35 (0.95, 1.90) |
|  | Q4 | 1.56 (1.11, 2.20)* |
|  | *P*-trend | 0.014 |
| 1-PYR | Continuous | 1.11 (0.98, 1.24) |
|  | Q1 | Ref |
|  | Q2 | 1.29 (0.98, 1.71) |
|  | Q3 | 1.53 (1.10, 2.11)* |
|  | Q4 | 1.61 (1.12, 2.33)* |
|  | *P*-trend | 0.012 |

Note: Poisson regression models were applied. All results for PAH metabolites were ln-transformed. Model adjusted for creatinine, age, sex, race, educational level, marital status, PIR, NHANES cycle, cotinine, physical activity, and occupational exposure. Q1 to Q4 refer to the 1st through 4th quartiles of PAH metabolites. Ref, reference. **P* < 0.05; ***P* < 0.01; ****P* < 0.001.

**Table S8.** Relative risks (95% confidence intervals) for the associations between PAHs and MASLD (National Health and Nutrition Examination Survey, The United States, 2007–2012)

| Variables |  | *RR* (95% *CI*) |
| --- | --- | --- |
| 1-NAP | Continuous | 1.00 (0.92, 1.08) |
|  | Q1 | Ref |
|  | Q2 | 1.01 (0.76, 1.33) |
|  | Q3 | 0.91 (0.71, 1.18) |
|  | Q4 | 0.86 (0.63, 1.18) |
|  | *P*-trend | 0.269 |
| 2-NAP | Continuous | 1.18 (1.06, 1.32)** |
|  | Q1 | Ref |
|  | Q2 | 1.39 (0.92, 2.09) |
|  | Q3 | 1.91 (1.43, 2.53)*** |
|  | Q4 | 1.54 (1.06, 2.23)* |
|  | *P*-trend | 0.001 |
| 3-FLU | Continuous | 0.97 (0.87, 1.08) |
|  | Q1 | Ref |
|  | Q2 | 0.86 (0.63, 1.16) |
|  | Q3 | 1.04 (0.71, 1.53) |
|  | Q4 | 1.04 (0.69, 1.59) |
|  | *P*-trend | 0.534 |
| 2-FLU | Continuous | 1.07 (0.94, 1.23) |
|  | Q1 | Ref |
|  | Q2 | 1.04 (0.76, 1.42) |
|  | Q3 | 1.65 (1.09, 2.49)* |
|  | Q4 | 1.44 (0.93, 2.24) |
|  | *P*-trend | 0.066 |
| 1-PHE | Continuous | 1.12 (0.99, 1.27) |
|  | Q1 | Ref |
|  | Q2 | 1.24 (0.89, 1.72) |
|  | Q3 | 1.35 (0.92, 1.99) |
|  | Q4 | 1.48 (0.98, 2.22) |
|  | *P*-trend | 0.072 |
| 1-PYR | Continuous | 1.06 (0.93, 1.21) |
|  | Q1 | Ref |
|  | Q2 | 1.25 (0.93,1.68) |
|  | Q3 | 1.55 (1.09, 2.21)* |
|  | Q4 | 1.46 (0.96, 2.21) |
|  | *P*-trend | 0.066 |

Note: Poisson regression models were applied. All results for PAH metabolites were ln-transformed. Model adjusted for creatinine, age, sex, race, educational level, marital status, PIR, NHANES cycle, cotinine, physical activity, HEI-2015, and occupational exposure. Q1 to Q4 refer to the 1st through 4th quartiles of PAH metabolites. Ref, reference. **P* < 0.05; ***P* < 0.01; ****P* < 0.001.


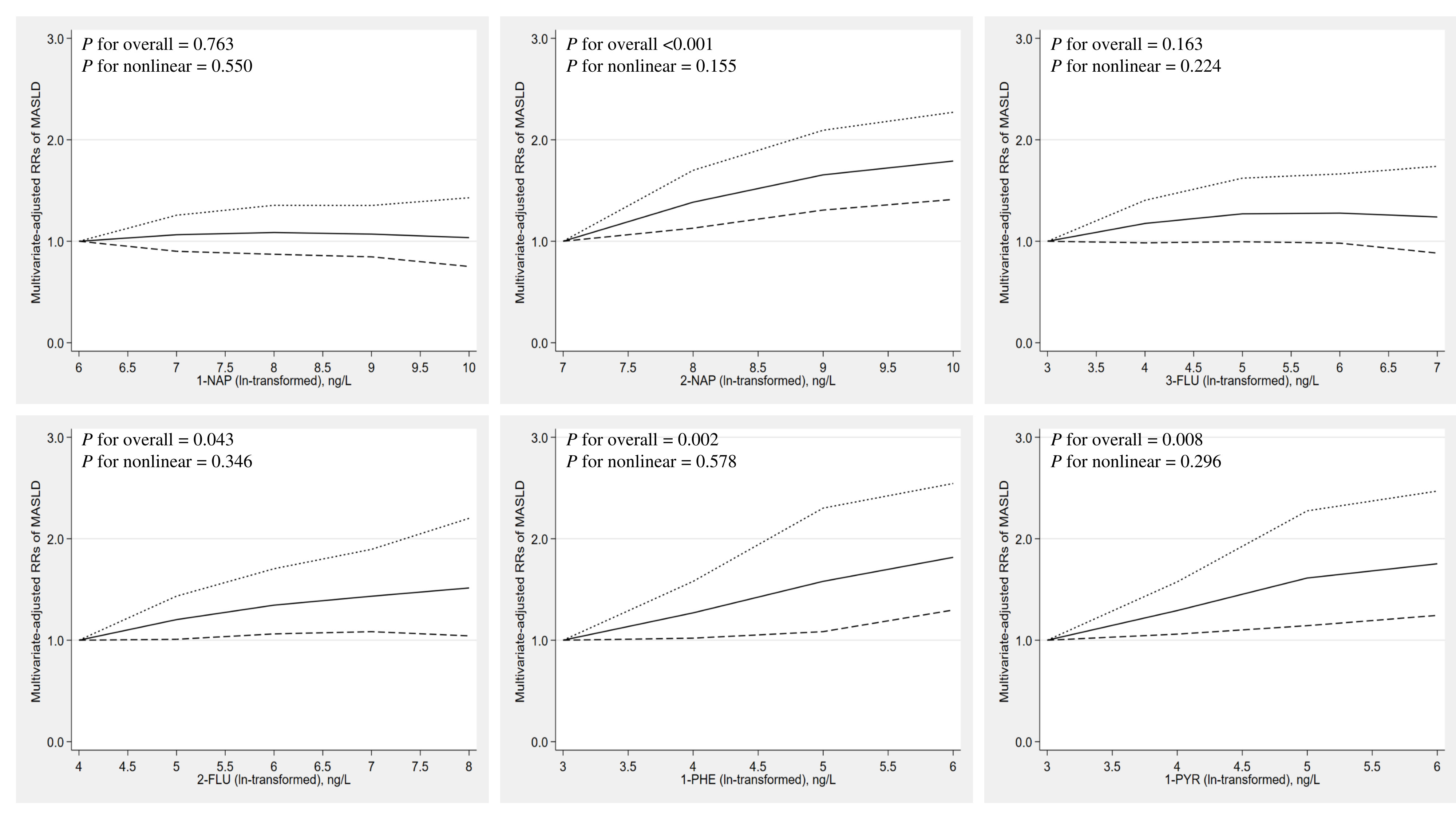


**Fig. S1** The results of restricted cubic spline (RCS) model of MASLD and individual PAH metabolites in full adjusted model.

Note: All results for PAH metabolites were ln-transformed. Covariates included creatinine age, sex, race, educational attainment, marital status, PIR, NHANES cycle, cotinine, and physical activity.

**
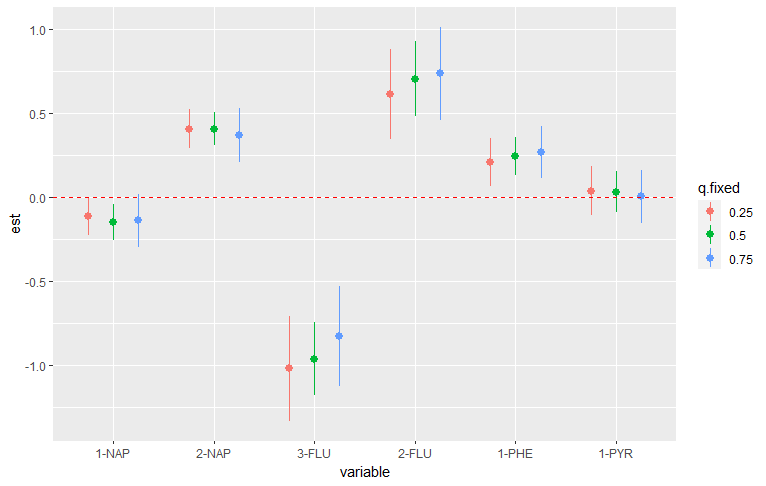
**

**Fig. S2** Associations of single PAHs with MASLD risk were estimated by BKMR models, when other all PAHs were held at their corresponding 25th (red), 50th (green), or 75th (blue) percentile, respectively.

Note: All results for PAH metabolites were ln-transformed. Covariates included creatinine age, sex, race, educational level, marital status, PIR, NHANES cycle, cotinine, and physical activity.


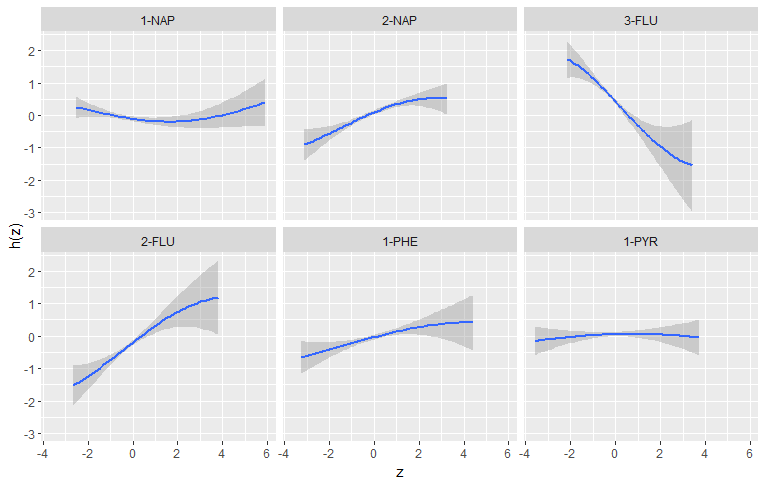


**Fig. S3** Univariate exposure-response functions (95% CI) for single PAHs when other PAHs fixed at the median.

Note: All results for PAH metabolites were ln-transformed. Covariates included creatinine age, sex, race, educational level, marital status, PIR, NHANES cycle, cotinine, and physical activity.
